# Increased expression of FAT4 suppress metastasis of lung adenocarcinoma through regulating MAPK pathway and associated with immune cells infiltration

**DOI:** 10.1002/cam4.4977

**Published:** 2022-06-30

**Authors:** Yue Ning, Yang Yang, Hongmei Zheng, Yuting Zhan, Hongjing Zang, Qiuyuan Wen, Jinwu Peng, Songqing Fan

**Affiliations:** ^1^ Department of Pathology, The Second Xiangya Hospital Central South University Changsha Hunan China; ^2^ Department of Pathology, Department of Pathology Xiangya Hospital of Central South University Changsha Hunan China

**Keywords:** bioinformatics, biomarker, FAT4 gene, lung cancer, MAPK

## Abstract

FAT4 is an extremely large atypical cadherin with crucial roles in the control of planar cell polarity (PCP) and regulation of the Hippo signaling pathway. Our study aims to clarify the FAT4 expression patterns, as well as the significance of FAT4 in predicting the prognosis and cancer immunity to non‐small cell lung cancer (NSCLC). FAT4 mRNA and protein expressions were both underregulated in NSCLC and associated with poor prognosis in both lung adenocarcinoma (LUAD) and lung squamous cell carcinoma (LUSC). In addition, overexpress FAT4 with jujuboside A (JUA) or knockdown FAT4 with siRNA regulated the metastasis of LUAD through MAPK pathways. Moreover, the FAT4 expression included multiple immunological components to promote an immunosuppressive tumor microenvironment (TME). Furthermore, a study of the TCGA‐LUAD cohort's DNA methylation results showed that most FAT4 DNA CpG sites were typically hypermethylated in NSCLC relative to the normal lung tissue. The DNA CpG sites cg25879360 and cg26389756 of FAT4 were found to be strongly associated with FAT4 expression in LUAD through the correlation study. In conclusion, this is the first to report the potential function of FAT4 in NSCLC. Hence, FAT4 could be used as a promising prognostic and immunological biomarker for NSCLC.

## Graphical abstract

Overexpression of FAT4 combined with jujuboside A (JUA) inhibits the metastasis of LUAD tumor cells through regulating MAPK pathways. Aberrant expression of FAT4 assocites with the tumor cell proliferation and immune infiltration in LUAD. Hypermethylated of CpG causes FAT4 expression decreased in LUAD.

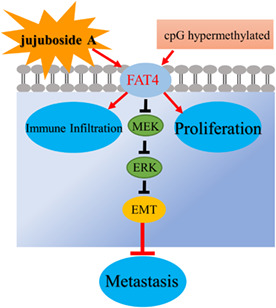

## 1 INTRODUCTION

Lung cancer is a common malignant tumor and a major cause of cancer‐related mortality worldwide.^1^ Non‐small cell lung cancer (NSCLC) accounts for over 85% of all lung cancer cases, with lung squamous cell carcinoma (LUSC) and lung adenocarcinoma (LUAD) being the two main subtypes.^2^ Surgical resection, chemotherapy, and radiotherapy are common treatments for early‐stage NSCLC, although all have been linked to unfavorable side effects.^3^ Recently, target therapy has received significant clinical attention as it is associated with fewer adverse effects and precisely targets tumor cells along with gene mutation of patients.^4^ However, the prognosis for NSCLC patients is still poor, and the five‐year survival rate of all advanced cases is only 15%–20%.^5^ It is thus necessary to identify better prognostic biomarkers for NSCLC.

FAT tumor suppressor homolog 4 (FAT4), FAT tumor suppressor homolog 4 (FAT4), a member of the FAT family that consists of FAT1‐4, encodes a single transmembrane protein containing extracellular cadherin repeats, a transmembrane domain, and a cytoplasmic domain.^6^ Increasing evidence suggests that the downregulation of FAT4 may be associated with the progression of several malignant carcinomas, including breast cancer, colorectal cancer, and gastric cancer.^7^, ^8^, ^9^, ^10^, ^11^, ^12^, ^13^ In endometrial cancer, the downregulation of FAT4 is due to the silencing of the deubiquitinating enzyme USP51, which exerts a tumor suppressor effect.^14^ In colorectal cancer, it was found that FAT4 regulated the PI3K/AKT/mTOR signaling pathway through modulating PI3K activity, preventing EMT, and inhibiting tumorigenesis.^15^ Furthermore, silencing of FAT4 stimulated proliferation, migration, and cell cycle progression in gastric cancer by modulating YAP nuclear translocation.^8^, ^10^, ^11^, ^13^ However, the role of FAT4 in NSCLC has not been investigated in great detail.

Herein, we explored the function of FAT4 in the pathophysiology of NSCLC by analyzing and investigating its expression patterns and thereby determining its potential in NSCLC prognosis.

## 2 MATERIALS AND METHODS

### 2.1 TCGA‐GTEx lung cancer cohort

The Cancer Genome Atlas (TCGA)—The Genotype‐Tissue Expression (GTEx) data was obtained from the University of California Santa Cruz Xena Browser.^16^, ^17^, ^18^ We used Excel to compile the data and discarded the recurrent tumors in favor of primary LUAD, primary LUSC, and normal tissue for future research.

### 2.2 Ethical statement

All research programs were admitted by the Ethics Review Committee of the Second Xiangya Hospital of Central South University (Scientific Research Ethics Committee, No. K021/2021), all samples for the research received written informed consent. When a juvenile received, the caretakers or guardian will sign a written consent represented the adolescent participant.

### 2.3 Patient cohorts and tissue microarrays (TMAs)

All samples were assembled from the blind for the reviewer. These NSCLC patients underwent definitive tumor resection in the thoracic surgery. At the time of the initial operation, no patient had administrated radiotherapy or chemotherapy. In this research, TMA technology was used to make high‐throughput NSCLC TMA following the technique described above. The microarray contained 241 LUAD, 239 LUSC, and 92 non‐cancer control normal lung tissues.

### 2.4 Survival analysis using KM‐plotter

KM‐Plotter,^19^ which contains the GEO and TCGA cohorts, was utilized to detect whether FAT4 is associated with the prognosis of NSCLC.

### 2.5 Gene set enrichment analysis (GSEA)

The co‐expression genes along with FAT4, which retrieved from the cBioPortal in the TCGA LUAD cohort.^20^, ^21^ GSEA was performed on these co‐expressed genes (*r* ≥ 0.35) in DAVID using the Kyoto Encyclopedia of Genes and Genomes (KEGG).^22^, ^23^ *p* < 0.05 value was considered enriched gene sets. GSEA was utilized to delve deeper into the pathways linked to FAT4 expression. Gene sets that met the *p* < 0.05 and FDR < 0.25 criteria were deemed significantly enriched.

### 2.6 Immunohistochemistry staining and scores

Immunohistochemical experiments were performed as mentioned previously.^24^ The dilution of the FAT4 primary antibody was 1:100 (Novus Biologicals, USA). Immunohistochemical staining was evaluated based on the staining intensity and blinded with clinical data. The assessment was performed using a semiquantitative approach that included the following: total score = intensity score × percentage score. The staining intensity of FAT4 was scored as 0 (negative), 1 (weak), 2 (medium), and 3 (strong). Furthermore, staining percentages were graded as follows: 4 (76%–100%), 3 (51%–75%), 2 (26%–50%), 1 (1%–25%), and 0 (0%). Staining scores ≤ 1 and >1 are considered to be the optimal critical levels of low/high expression of FAT4 protein, respectively.

### 2.7 Cell lines

The NSCLC cell lines (PC‐9, H460, H157, HCC827, H1299, and H1975) and normal bronchial epithelial cells (BEAS‐A2 and HBE) were gained from the ATCC (American type culture collection). Lung cancer cell lines were cultured with RPMI‐1640 medium (Thermo Fisher Scientific, USA) added 10% fetal bovine serum (Gibco, USA) in the incubator at 37°C, 5% CO_2_.

### 2.8 Transient transfection with siRNA

SiRNAs for FAT4 were synthesized by RiboBio, Inc. (Guangzhou, China). Cells were seeded into 6‐well plates at a density of 50% confluence for transfection. NSCLC cells were transfected with the siRNA or plasmid using Lipofectamine TM 3000 (Invitrogen Biotechnology, China) according to the manufacturer's protocol. After 48 to 72 h, cells were collected for further experiments.

### 2.9 CCK8 assays

The cytotoxic effects of jujuboside A (Selleck, USA) on NSCLC (PC‐9 and H157) cells were determined by the CCK8 assay. JUA treated the cell lines with the concentration from 0 to 500 μm for 48, 72, 96, and 120 h. CCK8 (Vazyme, China) solution was added to the wells. The OD value was checked by the microplate reader (Thermo, USA) at 450 nm after 2 h incubation. The IC50 values of JUA were calculated using GraphPad Prism software version 8.0.

### 2.10 Quantitative real time reverse transcription PCR (qRT‐PCR)

RNA was extracted from the cell lines by Trizol (Takara, Japan), and complementary DNA (cDNA) was synthesized with the PrimeScript RT reagent Kit (TaKaRa, Japan) according to the manufacturer's instructions. QRT‐PCR was performed in triplicate with the SYBR Premix ExTaq (TaKaRa, Japan).

### 2.11 Western blotting

Appropriate RIPA lysis buffer was added to the cells, then the proteins were collected and quantified with BCA protein assay kit (Vazyme, China). Protein heat denaturation was transferred to PAGE after SDS‐PAGE electrophoresis and immunodetected with the corresponding primary antibodies. Primary antibodies included anti‐FAT4 (Novus, USA), p‐MEK, MEK, Vimentin, Tublin, p‐ERK, ERK, E‐cadherin, N‐cadherin (CST, USA). Images were captured with ChemiDocTM CRSþ Molecular Imager (Bio‐Rad, USA).

### 2.12 Migration assays

Migration was measured with the Transwell (Millipore, USA) assay. Cells resuspended with 1640 were placed into the upper chamber, and the lower chamber filled in complete medium. After 16 h incubation, the chamber with the migrated cells in the bottom were fixed, stained, photographed, and cells were counted at three random airports for each filter.

### 2.13 Tumor‐immune infiltrating cells and immune genes related to FAT4 according to the tumor immune estimation resource database

The Tumor Immune Estimation Resource (TIMER) platform, an online platform that possesses gene‐specific correlation analyzes of tumor‐immune cells (TIICs), was utilized to investigate the correlation between all TIICs and FAT4 expression.^25^, ^26^, ^27^ Correlation modules applied to investigate the association between FAT4 and gene markers of tumor‐infiltrating immune cells. ESTIMATE algorithm calculated scores were downloaded from the ESTIMATE database.^28^ The pre‐calculated TCGA LUAD data from xCell databases have been downloaded xCell.^29^The enrichment score of 24 immune cells was evaluated based on ssGSEA algorithms applying the GSVA package v1.34.0. The connection between FAT4 and ESTIMATE scores, as well as 64 types of cells from the TCGA LUAD cohort, were subsequently studied with R Studio.

### 2.14 Statistical analysis

Considering data processing, GraphPad Prism 8.0 was utilized. Unless otherwise stated, all values in this study are expressed as mean ± standard deviation (SD). Two groups are contrasted in the independent survey. The student's *t*‐test was used when the SD of the two groups was equal, and the Student's *t*‐test with Welch's correlation was utilized when SD was different. One‐way ANOVA was employed when the variances in multigroup sample statistics were equal; otherwise, Welch's ANOVA was utilized. Bonferroni tests were performed on both ANOVAs. The correlations between FAT4 mRNA expression and copy number alterations (CNAs) were analyzed using Pearson's correlation analysis. Kaplan–Meier curves and log‐rank tests were hired to analyze the survival rates. ^*^ *p* < 0.05, ^**^ *p* < 0.01, and ^***^ *p* < 0.0001 represented the statistic difference level.

## 3 RESULTS

### 3.1 Analysis of FAT4 expression in multiple cancers

**FIGURE 1:**
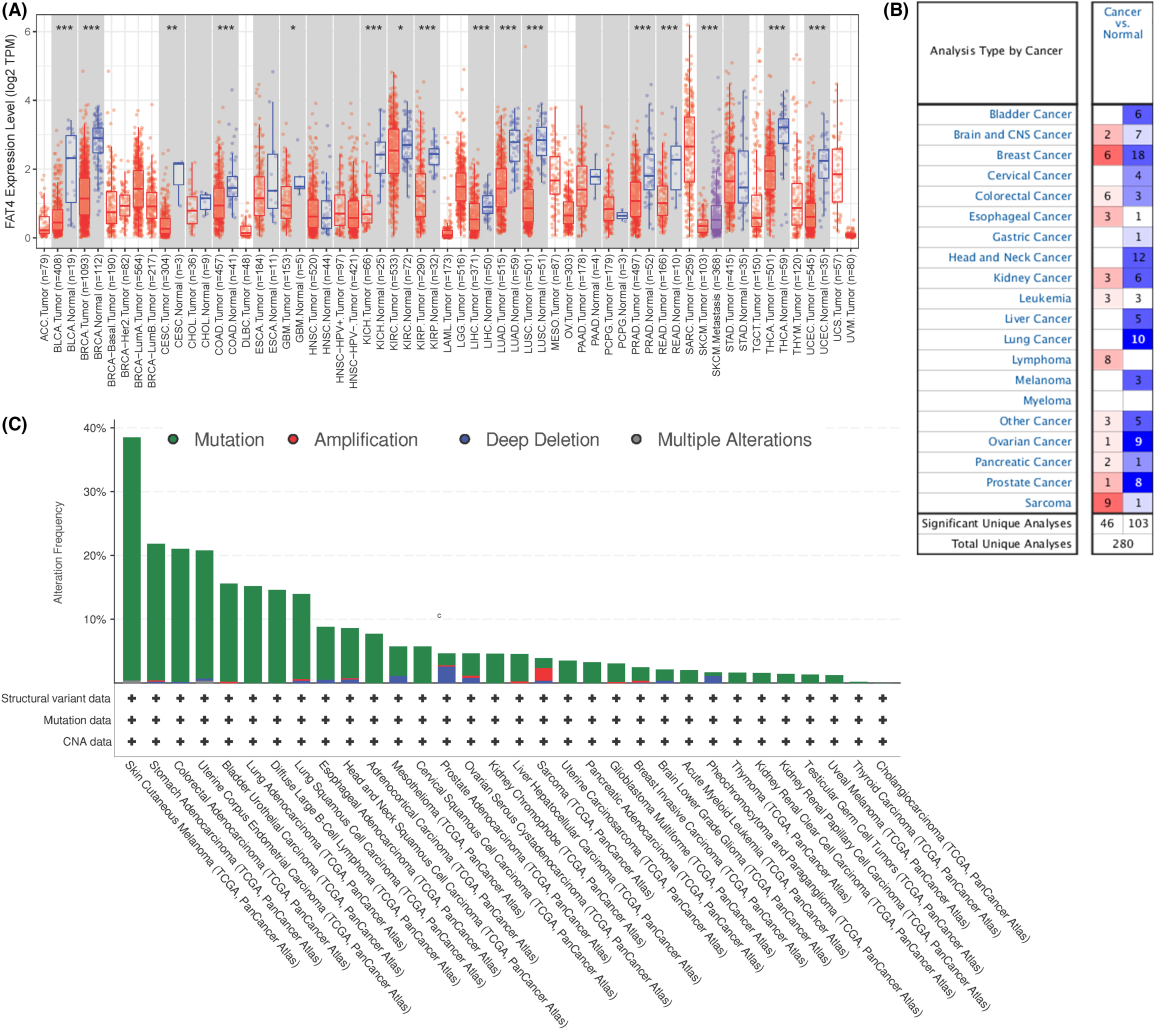
The aberrant expression of FAT4 in pan‐cancer. (A). The expression pattern of FAT4 in pan‐cancer by using tumor and compared normal tissue in TCGA cohort; (B). The summary of FAT4 expression in multiple cancer datasets in Oncomine. (C). The mutation landscape of FAT4 in pan‐cancer in TCGA cohort

Expression pattern of FAT4 in various cancers was first detected depending on TCGA‐GTEX datasets. As pictured in Figure 1A, FAT4 decreased in various tumor tissues, including ACC, BLCA, BRCA, CESC, COAD, ESCA, KICH, KIRP, LUAD, LUSC, OV, PRAD, READ, SKCM, THCA, UCEC, but elevated in GBM, LAML, LGG, PAAD, THCA, THYM. Subsequently, the ONCOMINE database, which visualizes GEO data was also exhibited the expression map of FAT4 in different kinds of cancers. As shown in Figure 1B, FAT4 down‐regulated in all the lung cancer datasets. Then, we further detected the mutation of FAT4 in multi‐kinds of cancer. The results suggested that the mutation of FAT4 over 10% in SKCM, STAD, COAD, UCEC, BLCA, LUAD, DLBL, LUSC (Figure 1C).

### 3.2 FAT4 level is notably decreased in NSCLC tissues and associated with poor prognosis

**FIGURE 2:**
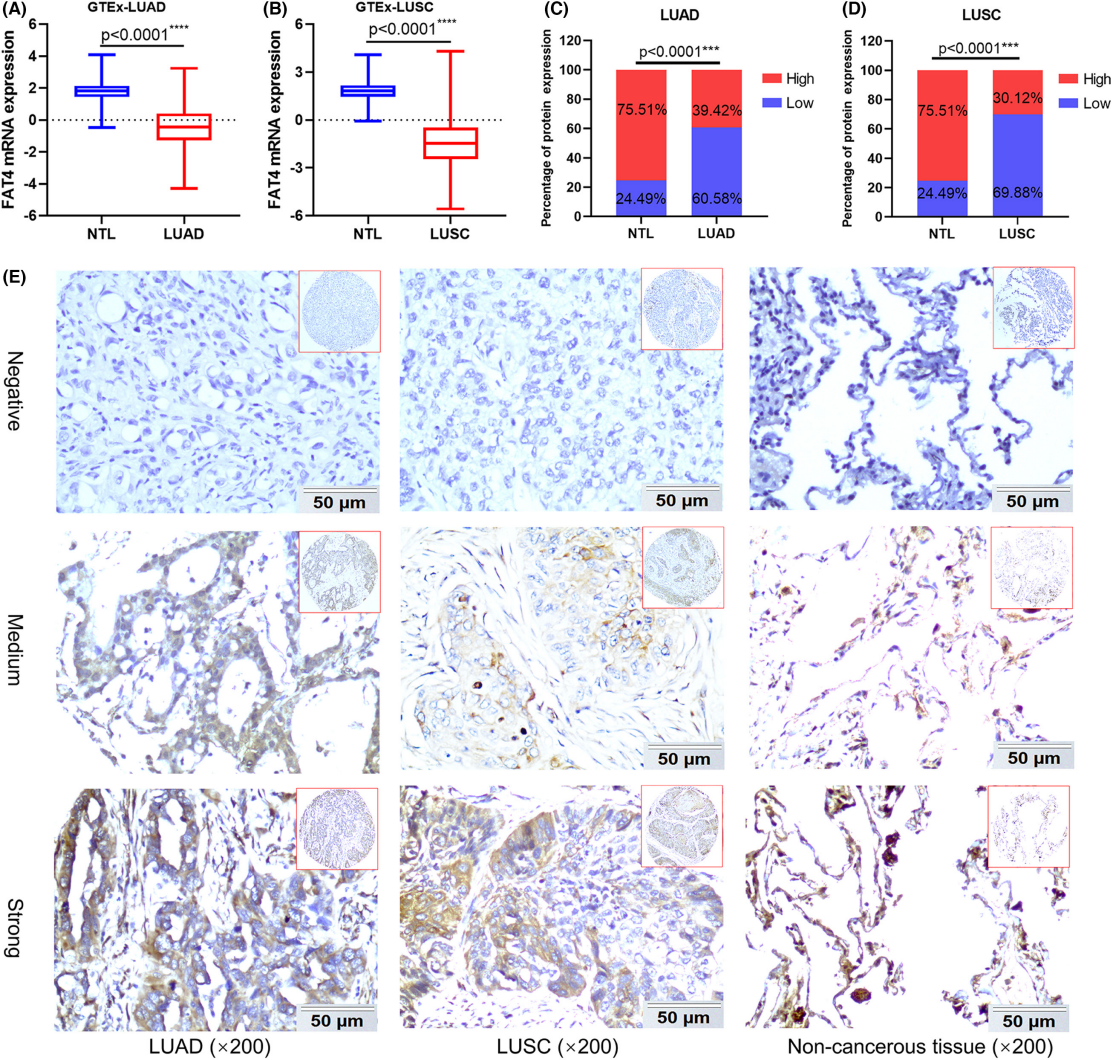
FAT4 expression is decreased in non‐small cell lung cancer (NSCLC) (A, B). FAT4 mRNA expression in LUAD and LUSC compared to that in normal lung tissues based on samples from the GTEx‐TCGA database. (C, D). FAT4 protein expression in LUAD and LUSC compared to that in non‐cancerous lung tissues based on tissue microarray by immunohistochemistry (IHC) (E). Representative IHC images show the differences in FAT4 protein expression between LUAD, LUSC, and non‐cancerous lung tissues

From the above results, FAT4 proved a significantly decreased expression in both LUAD and LUSC samples compared to normal lung tissue (Figure 2A–B). Furthermore, we performed an analysis of FAT4 with 27 NSCLC GEO cohorts. The results indicated FAT4 mRNA expression decreased in a majority of NSCLC cohorts (25/27) compared to the corresponding non‐tumor lung tissues (NTL), except for two cohorts (GSE20189 and GSE39345) (Table S1). Subsequently, FAT4 protein expression were evaluated in NSCLC patient samples by immunohistochemistry stain. We found decreased expression of FAT4 protein in tumor tissues compared to those in non‐cancerous lung tissues in LUAD and LUSC (Figure 2C–D). FAT4 protein expression was observed in the membrane and cytoplasm and was scarcely identified in the nucleus (Figure 2E).

**FIGURE 3:**
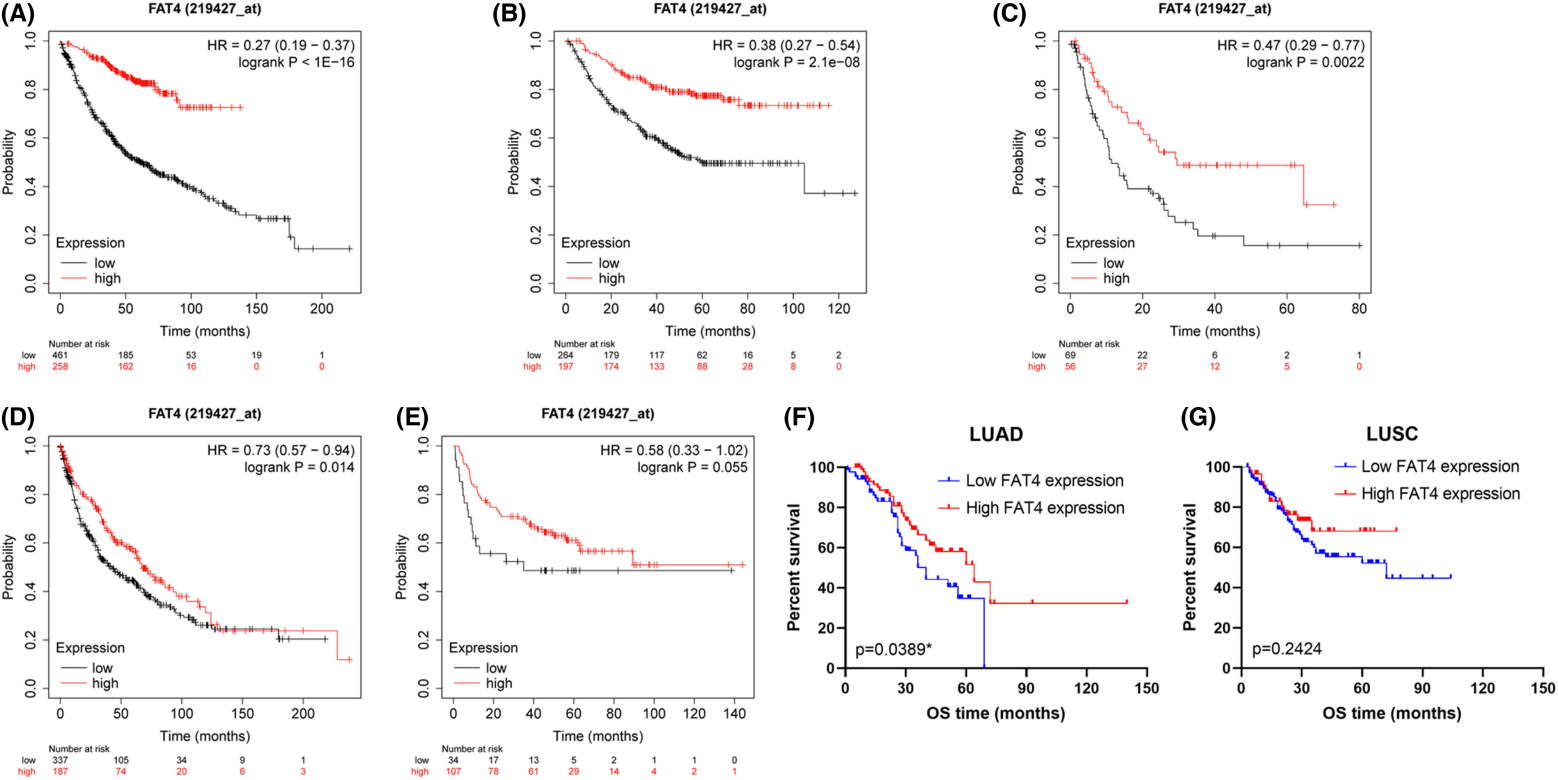
Decreased FAT4 expression confers a poor prognosis for LUAD. (A–C). OS, FPS, and PFS analysis of LUAD patients from KM‐plotter based on FAT4 expression. (D, E). OS, FPS, and PFS analysis of LUSC patients from KM‐plotter based on FAT4 expression. (F, G). Kaplan–Meier OS analysis of LUAD and LUSC patients fro TMAs based on FAT4 protein expression

The prognostic importance of decreased FAT4 expression were determined in NSCLC by the Kaplan–Meier plotter. Kaplan–Meier curves and log‐rank tests revealed that FAT4 mRNA expression was negatively connected with OS, FPS, and PFS in LUAD (Figure 3A–C), while only with OS in LUSC (Figure 3D–E). Besides, low expression of FAT4 protein was linked to poor prognosis for patients with LUAD (Figure 3F), but not LUSC (Figure 3G). Consequently, we concentrated on LUAD for further research.

### 3.3 Relationship between FAT4 and clinic pathological features in LUAD

The relationship of LUAD clinicopathology with FAT4 mRNA and protein was analyzed to figure out the function of FAT4 in LUAD development (Table 1). The results indicated that FAT4 expression decreased gradually following the tumor diameter, and that lymph nodes metastasis in the TCGA LUAD cohort were related to both mRNA and protein expression of FAT4. Furthermore, there was no significant associatiison among other features listed in Table 1. As a result, FAT4 promotes the development of the LUAD and lymph nodes metastasis.

**TABLE 1:** Correlation of FAT4 expression level with the clinicopathological features in LUAD

FAT4 mRNA				FAT4 protein				
Variable	Number	FAT4	*p* value	Variable	Number	Low	High	*p* value
Age (years)								
≤65	148	7.63 ± 1.35		≥55	126	77 (37.75%)	49 (24.02%)	
>65	191	7.84 ± 1.22	0.1942	<55	78	47 (23.04%)	31 (15.20%)	0.9033
Gender								
Female	171	8.00 ± 1.23		Female	90	63 (28.13%)	27 (12.05%)	
Male	168	7.52 ± 1.29	0.0005**, ***	Male	114	76 (33.93%)	58 (33.93%)	0.0446*
T								
≤3 cm	102	8.10 ± 1.32		≤3 cm	40	30 (14.71%)	10 (4.90%)	
>3 cm	237	7.62 ± 1.24	0.0015**	>3 cm	164	94 (46.08%)	70 (34.31%)	0.0472*
N								
N0	213	7.88 ± 1.30		N0	68	46 (21.30%)	22 (10.19%)	
N1‐3	126	7.58 ± 1.23	0.0398*	N1‐3	136	78 (36.11%)	58 (32.41%)	0.0391*
M				Pathological degree				
M0	317	7.77 ± 1.26		Well/moderated	127	77 (37.75%)	50 (24.51%)	
M1	22	7.66 ± 1.54	0.6905	Poor	77	47 (23.04%)	30 (14.71%)	0.9537
Clinical stages								
I–II	256	7.79 ± 1.26		I–II	117	70 (34.83%)	47 (23.38%)	
III–IV	83	7.68 ± 1.33	0.4907	III–IV	97	54 (26.87%)	43 (14.93%)	0.5215

*Note*: Abbreviation: LUAD, lung adenocarcinoma.

* *p* < 0.05

** *p* < 0.01

*** *p* < 0.001.

### 3.4 FAT4 suppressed the proliferation and metastasis of NSCLC

**FIGURE 4:**
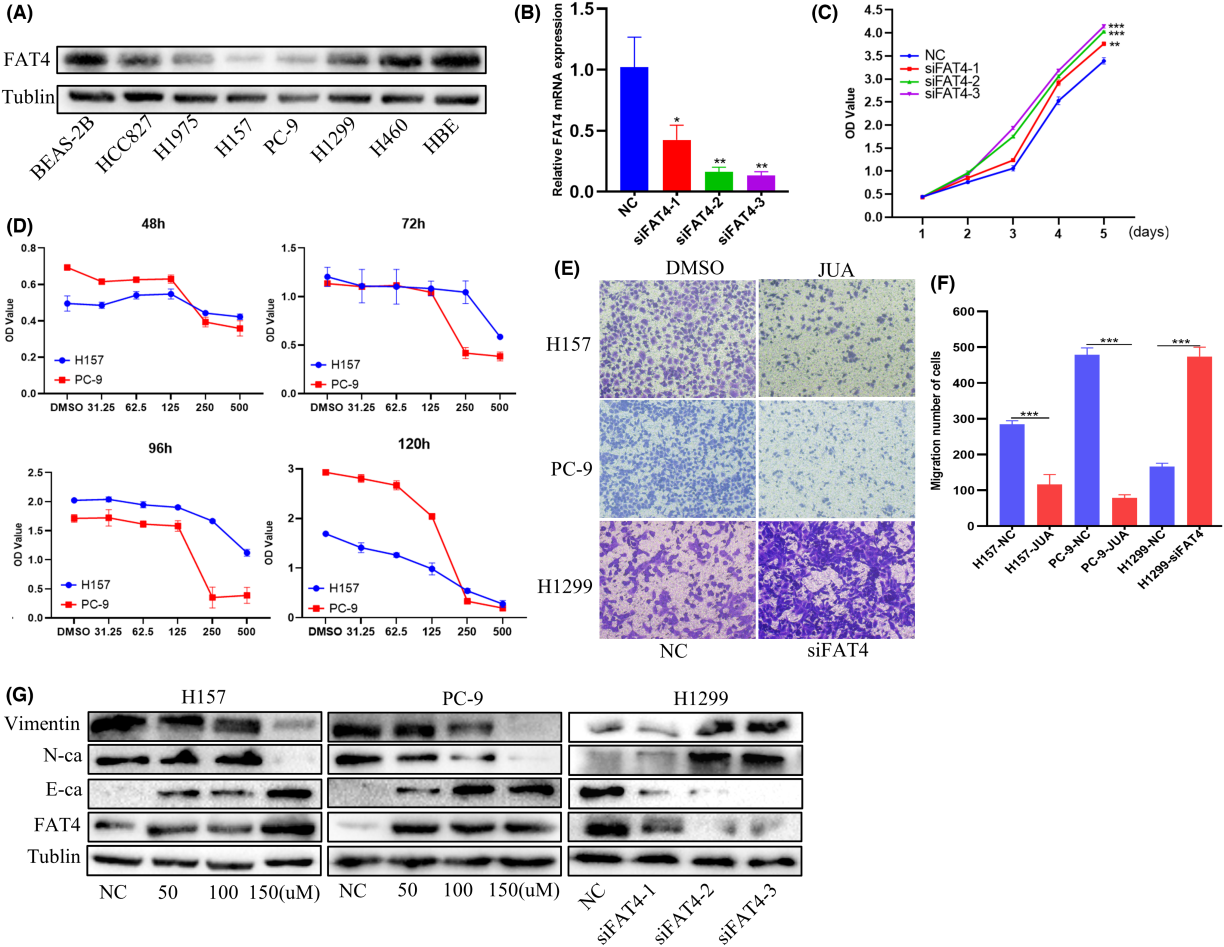
Overexpressed FAT4 with JUA or knockdown FAT4 with siRNA regulate proliferation and migration of lung adenocarcinomas (A). Protein expression of FAT4 in NSCLC cell lines and bronchial epithelial cells. (B). mRNA expression of FAT4 in H1299 transfected with FAT4. (C). CCK8 analysis of H1299 with FAT4 knockdown. (D). Cell viabilities of H157 and PC‐9 cells by CCK8 assay after being treated with JUA (0–500 μM) for 48, 72, 96, and 120 h, respectively. (E,F). Transwell assays of FAT4 with JUA or siRNA in lung adenocarcinomas cells. (G). Western blot analysis of EMT related protein expression levels in lung adenocarcinoma cells

To clarify the role of FAT4 in LUAD, protein expression of FAT4 was examined in lung cancer cells. FAT4 showed decreased expression in HCC827, H1975, H157, PC‐9, H1299, and H460 compared to the two normal bronchial epithelial cells, BEAS‐A2 and HBE (Figure 4A). And the H157 and PC‐9, which suggested the lowest protein expression among six NSCLC were selected for further study. JUA is an herbal traditional Chinese medicine that has been shown to overexpress FAT4 by direct combining with it.^30^ Treated H157 and PC‐9 with JUA increased the FAT4 expression. The CCK8 assays suggested that overexpressed FAT4 with JUA inhibited the proliferation of H157 and PC‐9 in a time and dose‐dependent manner, in which the IC50 for each are 120 and 149.7 μm at 5 days treatment (Figure 4D). Reversely, we still transfected siRNA in H1299 to the decreased FAT4. The siRNA blocked the FAT4 expression successfully (Figure 4B,G). CCK8 results demonstrated lessen FAT4 promotes the growth of H1299 (Figure 4C). Transwell migration assays suggested that JUA suppressed the migration ability of H157 and PC‐9 (Figure 4E–F), while decreased FAT4 promote metastasis of H1299. In addition, overexpressed FAT4 with JUA induces epithelial‐mesenchymal transformation (EMT) feature, accompanied by decreased N‐cadherin, Vimentin, and increased E‐cadherin (Figure 4G). Reduced FAT4 expression displayed the reverse results.

### 3.5 FAT4 suppressed the metastasis of NSCLC by inhibiting MAPK pathway

**FIGURE 5:**
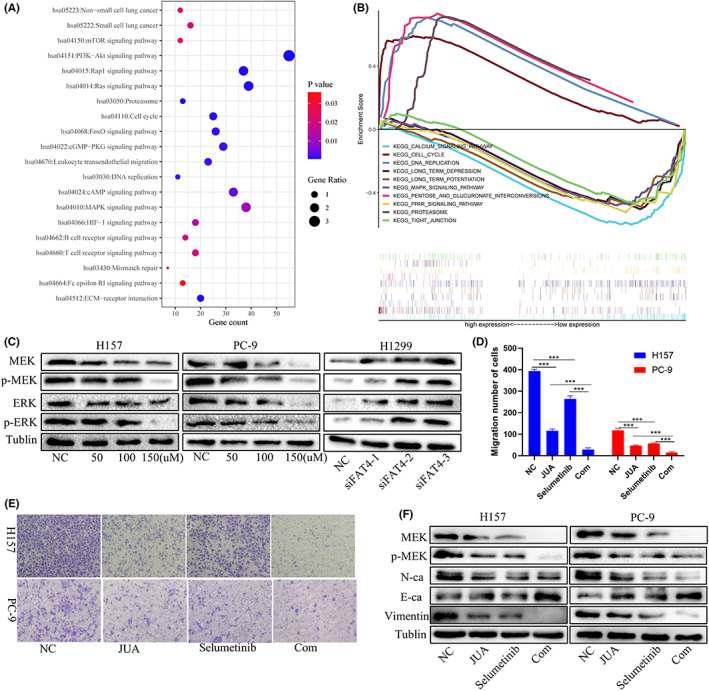
Decreased FAT4 promotes metastasis of LUAD via modulating MAPK pathway (A). Gene Set Enrichment Analysis employed co‐expression genes based on KEGG pathway database analysis. (B). GSEA analysis manifests potential pathways that implicate FAT4. (C). Western Blot results validated overexpressed or knockdown FAT4 regulate the activation of MAPK pathway. (D, E). Transwell assays for MEK inhibitor, Selumetinib in FAT4 overexpressed lung adenocarcinomas cells. (F). Western Blot for EMT related protein expression of H157 and PC‐9 cells with MEK inhibited

The crucial role of FAT4 in NSCLC metastasis prompted us to investigate the mechanisms of FAT4 how to inhibit growth and metastasis of NSCLC cells. To grope the downstream pathways, which FAT4‐mediated NSCLC metastasis, GSEA was performed to explore the biological activities and possible pathways linked with FAT4. The FAT4 co‐expression genes were identified via cBioPortal. Through DAVID analysis, we received the KEGG pathways for FAT4. Based on KEGG pathway results, FAT4 regulates the cell cycle, mTOR, and HIF‐1 signaling pathways (Figure 5A). The processes related to FAT4 expression were also confirmed with GSEA. According to our research, FAT4 may joined in the regulation cell cycle, MAPK, and PPAR pathways (Figure 5B). Comparing the two enrichment results, both of them contained cell cycle and MAPK regulation. According to the GSEA results, the relationship between FAT4 and MAPK signaling pathway was examined. When the FAT4 overexpressed, the MEK and ERK, along with p‐MEK and p‐MEK were significantly downregulated (Figure 5C), knockdown of FAT4 revealed the opposite results. Transwell migration assay proved that the combined treatment with MEK‐specific inhibitor, selumetinib, and JUA significantly suppressed the migration of H157 and PC‐9 (Figure 5D,E). Additionally, N‐cadherin, Vimentin were downregulated while upregulated E‐cadherin in H157 and PC‐9 treated with JUA and MEK inhibitor, Selumetinib (Figure 5F). The above results proved that overexpressed FAT4 lessens metastasis of NSCLC via regulating MAPK pathway.

### 3.6 Relationship between FAT4 with the proportion of TIICs

**FIGURE 6:**
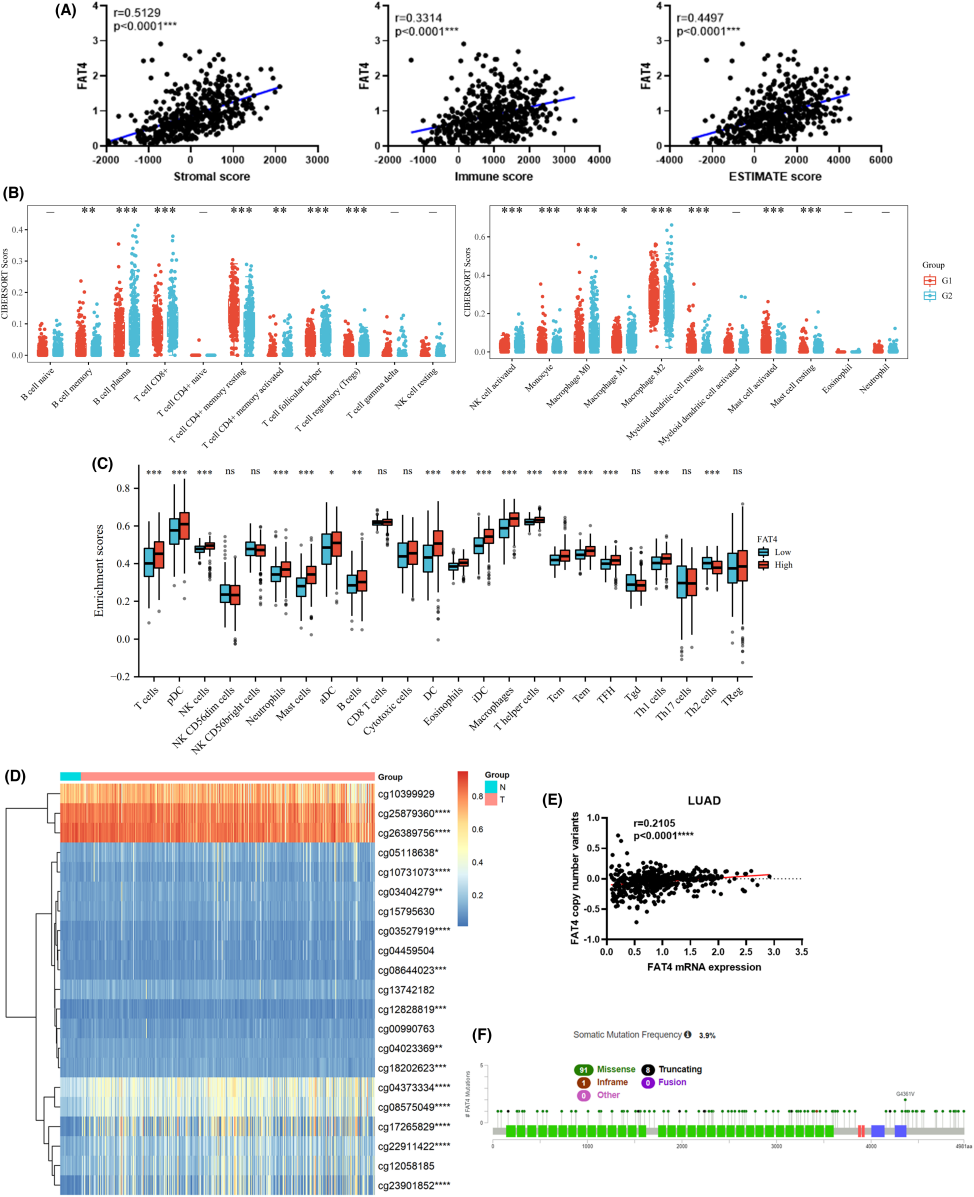
Correlation between FAT4 mRNA expression and its DNA methylation, copy number variation, and somatic mutation in TCGA LUAD patients (A). FAT4 expression positively correlated with the immune score, stromal score, and estimate score in LUAD patients. (B) Infiltration fraction between FAT4 expression and 22 immune cells in TCGA‐LUAD cohort according to the CIBERSORT algorism. (C) Boxplots pictured FAT4 expression and the score of 28 immune cells in TCGA‐LUAD datasets with ssGSEA algorism. (D). FAT4 DNA methylation in LUAD and healthy lung tissue. (E). Correlation study between FAT4 mRNA expression and its DNA copy number alteration in LUAD. (F). FAT4 somatic mutation map in LUAD

From the Gene enrichment analysis, we found that FAT4 connected to several immune‐related pathways, such as T/B cell receptor, Fc epsilon RI signaling pathways. Therefore, xCells data was applied to validate the relationship between FAT4 expression and the immune components, which include 64 immune cell types profiles in LUAD, and analyzed the proportion of tumor‐infiltrating immune subtypes. First, we looked for an association between FAT4 expression and immunological scores. FAT4 was shown to have a strong connection with Stromal Score, Immune Score, and ESTIMATE score in LUAD patients at the same time (Figure 6A). Meanwhile, we examined the relationship between FAT4 and 64 non‐carcinogenic cells by xCell to identify the key components that participated in FAT4 related immunological processes. We demonstrated that 49 cell kinds were associated with FAT4, among which 31 kinds were positively connected, whereas 18 kinds were negatively connected (Table 2). Likewisely, the CIBERSORT algorithm results suggested that FAT4 expression associated with infiltrating B cell memory and plasma cells, CD8 T^+^ cells, T cells CD4^+^ memory resting, T cells CD4^+^ memory activated, T cells follicular helper, Tregs, NK cell activated, Monocyte, Macrophage M0, M1, M2, Myeloid dendritic cells resting, activated and resting mast cells in the TCGA‐LUAD. Besides, ssGSEA displayed that activated T and B cells, T helper cells, Tcm, Tem, Tfh, Th1 cells, DC, pDC, aDC, and iDC, NK cells, Macrophages, Neutrophils, Eosinphils and Mast cells were significantly enriched in the FAT4 low expression group of TCGA‐LUAD cohorts. Interestingly, the cellular components that existed a strong correlation with FAT4, such as CD4+ and CD8+ naïve T cells, CD4+ T cells, B cells, and Th1/2 cells, have proved to play crucial roles in the LUAD TME. We also validated the relationship between FAT4 and diverse immune marker sets of various tumor‐infiltrating immune cells in LUAD. The analysis suggested that FAT4 expression was strongly associated with the levels of a large part of marker sets of T cells, B cells, monocytes, TAMs, M1/2 macrophages, neutrophils, dendritic cells, Th2, Th9, and Tregs in LUAD (Table S2). Surprisingly, CTLA4 and TIM‐3, which are important genes in mediating T cell exhaustion, have a clear positive association with FAT4 expression, implying that low FAT4 expression is important in modulating T cell exhaustion. All of these confirmed that FAT4 is directly associated with tumor‐infiltrating immune cells in LUAD, suggesting that FAT4 is essential for immune escape and influenced the immune activity in TME of LUAD.

**TABLE 2:** Correlation between FAT4 and 64 types of non‐cancerous cells in LUAD

xCells	Category	Pearson's *r* (95% CI)	*p* value
B cells	Lymphoids	0.21 (0.12–0.29)	<0.0001***
CD4+ memory T cells	Lymphoids	0.09 (0.00–0.18)	0.0428*
CD4+ naive T cells	Lymphoids	0.34 (0.26–0.41)	<0.0001***
CD4+ T cells	Lymphoids	0.32 (0.21–0.42)	<0.0001***
CD4+ Tcm	Lymphoids	−0.10 (−0.21–0.022)	0.1115
CD4+ Tem	Lymphoids	−0.14 (−0.24–0.03)	0.0091*
CD8+ naive T cells	Lymphoids	−0.23 (−0.31–0.14)	<0.0001***
CD8+ Tcm	Lymphoids	−0.09 (−0.21–0.02)	0.1115
CD8+ Tem	Lymphoids	0.04 (−0.07–0.14)	0.4958
CD8 + T cells	Lymphoids	0.09 (−0.00–0.18)	0.0620
Class switched memory B cells	Lymphoids	0.12 (0.026–0.20)	0.0112*
Memory B cells	Lymphoids	0.10 (−0.00–0.21)	0.0548
Naive B cells	Lymphoids	0.15 (0.05–0.24)	0.0019**
NK cells	Lymphoids	−0.00 (−0.11–0.11)	0.9763
Natural killer T cells (NKT)	Lymphoids	−0.17 (−0.26–0.085)	<0.0001***
Plasma cells	Lymphoids	−0.24 (−0.32–0.15)	<0.0001***
pro B cells	Lymphoids	−0.22 (−0.33–0.11)	0.0002**
Tgd cells	Lymphoids	−0.14 (−0.26–0.02)	0.0221*
Th1 cells	Lymphoids	−0.51 (−0.58–0.45)	<0.0001***
Th2 cells	Lymphoids	−0.28 (−0.36–−0.19)	<0.0001***
Tregs	Lymphoids	0.12 (0.027–0.21)	0.0115*
Activated dendritic cells (aDC)	Myeloids	0.16 (0.06875–0.2401)	0.0005**
Basophils	Myeloids	‐0.19 (−0.28–0.10)	<0.0001***
Conventional dendritic cells (cDC)	Myeloids	0.34 (0.26–0.41)	<0.0001***
Dendritic cells (DC)	Myeloids	0.31 (0.23–0.39)	<0.0001***
Eosinophils	Myeloids	0.21 (0.13–0.30)	<0.0001***
Immature DC (iDC)	Myeloids	0.26 (0.18–0.34)	<0.0001***
Macrophages	Myeloids	0.07 (−0.02–0.16)	0.1148
Macrophages M1	Myeloids	0.013 (−0.07–0.10)	0.7544
Macrophages M2	Myeloids	0.16 (0.076–0.25)	0.0003**
Mast cells	Myeloids	0.51 (0.45–0.58)	<0.0001***
Monocytes	Myeloids	0.22 (0.14–0.31)	<0.0001***
Neutrophils	Myeloids	0.04 (−0.05–0.13)	0.3667
Plasmacytoid dendritic cells (pDC)	Myeloids	−0.00 (−0.09–0.08)	0.9104
Astrocytes	Others	0.1218 (0.01766–0.2234)	0.0221*
Epithelial cells	Others	−0.19 (−0.27–0.09)	<0.0001***
Hepatocytes	Others	−0.10 (−0.18–0.00)	0.0325*
Keratinocytes	Others	−0.11 (−0.20–0.03)	0.0110*
Melanocytes	Others	0.032 (−0.06–0.12)	0.4808
Mesangial cells	Others	0.22 (0.14–0.30)	<0.0001***
Myocytes	Others	0.23 (0.15–0.31)	<0.0001***
Neurons	Others	−0.011 (−0.10–0.08)	0.8107
Sebocytes	Others	−0.22 (−0.30–0.14)	<0.0001***
Common lymphoid progenitors (CLP)	Stem cells	−0.39 (−0.46–0.31)	<0.0001***
Common myeloid progenitors (CMP)	Stem cells	0.18 (0.06–0.29)	0.0028**
Erythrocytes	stem cells	−0.094 (−0.20–0.01)	0.0748
Granulocyte‐macrophage progenitor (GMP)	Stem cells	0.38 (0.28–0.47)	<0.0001***
Hematopoietic stem cells (HSC)	Stem cells	0.50 (0.43–0.56)	<0.0001***
Megakaryocytes	Stem cells	0.43 (0.37–0.51)	<0.0001***
Megakaryocyte‐erythroid progenitors (MEP)	Stem cells	−0.46 (−0.53‐‐0.39)	<0.0001***
Multipotent progenitors (MPP)	Stem cells	−0.21 (−0.32‐‐0.11)	0.0001***
Platelets	Stem cells	0.15 (0.024–0.26)	0.0192*
Adipocytes	Stromal cells	0.2750 (0.1922–0.3539)	<0.0001***
Chondrocytes	Stromal cells	0.47 (0.40–0.54)	<0.0001***
Endothelial cells	Stromal cells	0.40 (0.32–0.47)	<0.0001***
Fibroblasts	Stromal cells	0.60 (0.54–0.65)	<0.0001***
ly Endothelial cells	Stromal cells	0.33 (0.25–0.40)	<0.0001***
Mesenchymal stem cells (MSC)	Stromal cells	−0.08 (−0.17–0.011)	0.0870
mv Endothelial cells	Stromal cells	0.22 (0.14–0.30)	<0.0001***
Osteoblast	Stromal cells	−0.43 (−0.50–0.36)	<0.0001***
Pericytes	Stromal cells	0.24 (0.15–0.32)	<0.0001***
Preadipocytes	Stromal cells	0.39 (0.26–0.51)	<0.0001***
Skeletal muscle	Stromal cells	−0.04 (−0.14–0.062)	0.4358
Smooth muscle	Stromal cells	−0.36 (−0.43–0.28)	<0.0001***

*Note*: Abbreviation: LUAD, lung adenocarcinoma.

* *p* < 0.05.

** *p* < 0.01.

*** *p* < 0.001.

### 3.7 Aberrant FAT4 mRNA expression is associated with DNA copy number alteration and hypermethylation in LUAD

Epigenetic modulation is a well‐known method of controlling gene expression. As a result, we investigated FAT4 copy number alteration (CNAs), methylation, and somatic mutations in LUAD. With the exception of cg10399929, cg15795630, cg04459504, cg13742182, cg00990763, and cg12058185, most of the methylation sites in LUAD (6/21) were substantially altered relative to those in normal tissues (Figure 6B). We also examined the relationship of various methylation sites to the expression of FAT4 mRNA. The cg25879360 and cg26389756 sites demonstrated a positive association with FAT4 mRNA expression (Table S3), indicating that FAT4 expression may be diminished. Furthermore, the findings of our study indicated a highly positive association between CNA and FAT4 mRNA in LUAD (Figure 6C). The majority of FAT4 somatic mutations were missense mutations, which may lead to irregular FAT4 expression (Figure 6D). Unfortunately, FAT4 mutations are scattered and no hotspots have been established.

## 4 DISCUSSION

FAT4 belongs to the fat cadherin family, which are calcium‐dependent cell adhesion proteins.^6^ Despite identifying the role and interaction proteins of FAT4 in vertebrates, the role of FAT4 in tumors has not been well studied, especially in lung cancer. The function of FAT4 and its associated signaling mechanisms in LUAD tumorigenesis was examined in this study.

In previous studies, bioinformatics analysis indicated lower FAT4 expression in ovarian cancer tissues compared to that in normal tissues.^9^ Immunohistochemical analysis clarified lower FAT4 expression in gastric,^8^ colorectal,^15^ and endometrial cancer.^14^ In addition, downregulation of FAT4 is correlated with lymph node metastasis and poor prognosis in endometrial cancer and gastric cancer.^8^, ^14^ Our analysis in NSCLC first demonstrated that FAT4 was decreased in TCGA, GEO NSCLC samples, and our TMA samples and positively correlated with OS, FPS, and PFS. Besides, our study found that FAT4 is active in lymph node metastasis and proliferation in clinical LUAD samples, and FAT4 inhibits the proliferation and metastasis of NSCLC in vitro, which is consistent with the KEGG gene enrichment study, which revealed that FAT4 is joined in the modulation of cell cycle and DNA replication, all of which are linked to tumor proliferation. Previous findings also proved FAT4 inhibits proliferation and metastasis in breast cancer,^7^ endometrial cancer,^14^ and gastric cancer cell lines.^8^, ^10^, ^13^, ^31^ FAT4 knockdown in endometrial^14^ and gastric^10^ cancer cells promote cell cycle progression. Previous reports on the signaling pathways regulated by FAT4 in cancers have mostly focused on PI3K/AKT/mTOR^15^ Hippo pathway^10^ and β‐catenin/Wnt.^32^ Here, we proved FAT4 modulating NSCLC metastasis by MAPK pathways, which consistent with GSEA prediction.

While FAT4 has been documented in several cancers of its suppressor role, the function of FAT4 in tumor immunity has not been investigated. Here, we found that FAT4 strongly connected with immune score, stromal score, and microenvironment score, and correlated with infiltration of NK cells, Macrophages, and T/B cells, which considering to poor prognosis for many cancers. Overall, our research was administrated to understand the possible function of FAT4 on antitumor immunotherapy response and the prognosis, but all of these results need further confirmation.

Aberrant gene expression is normally followed by atypical cancer cell activity due to genetic and epigenetic alterations, such as somatic mutations, DNA methylation, and CNA. Aberrant methylation of the FAT4 promoter has been documented in gastric^8^, ^11^ and breast cancer.^12^ The epigenetic modification of FAT4 in the TCGA LUAD dataset was examined here. Abnormal FAT4 expression may cause by CNA and DNA methylation (cg25879360 and cg26389756). Although the somatic mutation of FAT4 in LUAD patients is heterogeneous and lacks a hot spot, it could inactivate FAT4 expression.

## 5 CONCLUSIONS

In summarize, our study is the first to reveal FAT4 suppressed metastasis of LUAD through MAPK pathways. We discovered that FAT4 expression levels were lower in NSCLC and linked to OS, FPS, and PPS in LUAD. We also discovered that CNA and DNA methylation were related to aberrant FAT4 expression in LUAD. Unfortunately, our analysis was limited by the absence of in vivo validation. Although multiple pathways were enriched by the GSEA analysis, our analysis implicated only that FAT4 affects proliferation and migration through the MAPK pathway.

## AUTHOR CONTRIBUTION

Yue Ning, jinwu Peng, and Songqing Fan conceived the research. Yang Yang wrote the manuscript. Hongmei Zheng collected the literature. Yuting Zhan, Hongjing Zang, Qiuyuan Wen made significant revisions to the manuscript.

## CONFLICT OF INTEREST

The authors declare no conflict of interest.

## ETHICS STATEMENT

All experiments were approved by the Ethics Committee of Medicine and Animal Ethics Committee of the Second Xiangya Hospital, Central South University. Informed consent was obtained from all patients.

## Supporting information


Table S1
Table S2
Table S3

## Data Availability

The datasets used during the present study are available from the corresponding author upon reasonable request. Data were obtained from The Cancer Genome Atlas (TCGA; https://portal.gdc.cancer.gov), cBioportal (https://www.cbioportal.org/), the University of California Santa Cruz Xena Browser (https://xenabrowser.net), TIMER (http://timer.cistrome.org/), GEO (https://www.ncbi.nlm.nih.gov/geo/) and Oncomine (https://www.oncomine.org/resource/login.html).
