# Supplementary material for: Increased expression of FAT4 suppress metastasis of lung adenocarcinoma through regulating MAPK pathway and associated with immune cells infiltration
Source: Cancer Med. 2022 Jun 30;12(2):1616–29. doi: 10.1002/cam4.4977 (PMC9883428; doi:10.1002/cam4.4977)
Supplement: Supplementary file 1 — Table S1 Table S2 Table S3 [file CAM4-12-1616-s001.docx]

**Supplement**

**Table S1. Expression of FAT4 in TCGA and GEO NSCLC datasets**

| **Author** | **Source** | **Country** | **Series** | **Cancer** | | **Normal** | | **P value** |
| --- | --- | --- | --- | --- | --- | --- | --- | --- |
|  |  |  |  | N | Mean ± SD | N | Mean ± SD |  |
| NSCLC | | | | | | | | |
| Ramos AS | Tissue | Spain | GSE18842 | 45 | 5.40±0.57 | 46 | 4.37±0.82 | <0.0001^***^ |
| Philipsen S | Tissue | Netherlands | GSE19188 | 65 | 0.96±0.50 | 91 | -0.70±0.92 | <0.0001^***^ |
| Rotunno | Blood | USA | GSE20189 | 80 | 3.65±0.13 | 73 | 3.66±0.13 | 0.5944 |
| Li-Jen S | Tissue | Taiwan | GSE27262 | 25 | 1.37±0.39 | 25 | 0.65±0.37 | <0.0001^***^ |
| Sophie | Tissue | France | GSE30219 | 14 | 6.98±0.46 | 293 | 5.41±0.92 | <0.0001^***^ |
| Marquardt G | Tissue | USA | GSE31552 | 62 | 6.89±2.26 | 63 | 5.85±2.17 | 0.0095 |
| Meister M | Tissue | Germany | GSE33532 | 20 | 7.30±0.39 | 80 | 5.45±0.93 | <0.0001^***^ |
| Chen Y | Blood | Taiwan | GSE39345 | 20 | 1.64±1.44 | 32 | 1.63±1.52 | 0.7838 |
| Kastner S | Tissue | Austria | GSE40275 | 43 | 6.61±0.37 | 41 | 5.36±1.29 | <0.0001^***^ |
| Seo J | Tissue | USA | GSE40419 | 77 | 3.99±1.31 | 87 | 2.20±1.56 | <0.0001^***^ |
| Arima C | Tissue | Japan | GSE51852 | 4 | 2.76±0.34 | 49 | 1.10±1.35 | <0.0001^***^ |
| Khadijah | Tissue | USA | GSE101929 | 34 | 9.49±0.48 | 32 | 7.73±1.23 | <0.0001^***^ |
| LUAD | | | | | | | | |
| Su L | Tissue | Taiwan | GSE7670 | 27 | 8.46±0.50 | 27 | 7.35±0.97 | 0.0010^**^ |
| MariaTeresa | Tissue | USA | GSE10072 | 49 | 7.95±0.35 | 58 | 7.16±0.50 | <0.0001^***^ |
| David Q | Tissue | USA | GSE32665 | 92 | 8.22±0.70 | 87 | 7.55±0.31 | <0.0001^***^ |
| Suhaida A | Tissue | USA | GSE32863 | 58 | 6.70±0.11 | 58 | 6.98±0.12 | 0.5273 |
| Kadara H | Tissue | USA | GSE43458 | 30 | 9.24±0.42 | 80 | 7.94±0.79 | <0.0001^***^ |
| Feng L | Tissue | China | GSE43767 | 15 | 8.08±0.23 | 69 | 7.10±0.48 | <0.0001^***^ |
| Robles AI | Tissue | USA | GSE63459 | 32 | 7.04±0.12 | 33 | 6.94±0.15 | 0.0057^**^ |
| Gazdar A | Tissue | USA | GSE75037 | 83 | 4.39±0.54 | 83 | 4.16±0.55 | 0.0074^**^ |
| Rezzonico R | Tissue | France | GSE116959 | 11 | 7.25±0.64 | 57 | 6.07±0.59 | <0.0001^***^ |
| Mervi | Tissue | USA | GSE68645 | 19 | 5.98±0.29 | 443 | 7.20±0.83 | <0.0001^***^ |
| TCGA-LUAD | Tissue | TCGA | LUAD | 59 | 9.67±0.60 | 515 | 7.86±1.31 | <0.0001^***^ |
| Tzu-Pin | Tissue | Taiwan | GSE19804 | 60 | 7.87±0.67 | 60 | 7.09±1.02 | <0.0001^***^ |
| Kohno T | Tissue | Japan | GSE31210 | 20 | 10.82±0.34 | 226 | 9.97±0.93 | <0.0001^***^ |
| LUSC | | | | | | | | |
| Mascaux C | Tissue | USA | GSE33479 | 27 | 2.27±1.77 | 14 | 0.64±0.64 | 0.0001^***^ |
| TCGA-LUSC | Tissue | TCGA | LUSC | 51 | 9.85±0.57 | 502 | 6.96±1.40 | <0.0001^***^ |

Note: NSCLC, non-small cell lung cancer; LUAD, lung adenocarcinoma; LUSC, squamous lung carcinoma; ^*^ *P*<0.05; ^**^*P*<0.01; ^***^*P*<0.001.

**Table S2. Correlation analysis between FAT4 and relate genes and markers of immune cells in TIMER.**

| **Description** | **Gene markers** | **LUAD** | | | |
| --- | --- | --- | --- | --- | --- |
|  |  | None | | Purity | |
|  |  | Cor | P | Cor | P |
| CD8+ T cell | CD8A | 0.163 | 0.000^**^ | 0.062 | 0.168 |
|  | CD8B | 0.054 | 0.219 | -0.032 | 0.468 |
| T cell (general) | CD3E | 0.318 | <0.0001^***^ | 0.222 | <0.0001^***^ |
|  | CD3D | 0.203 | <0.0001^***^ | 0.083 | 0.064 |
|  | CD2 | 0.299 | <0.0001^***^ | 0.197 | <0.0001^***^ |
| B cell | CD19 | 0.208 | <0.0001^***^ | 0.112 | 0.013^*^ |
|  | CD79A | 0.210 | <0.0001^***^ | 0.121 | 0.001^*^ |
| Monocyte | CD86 | 0.389 | <0.0001^***^ | 0.318 | <0.0001^***^ |
|  | CSF1R | 0.511 | <0.0001^***^ | 0.454 | <0.0001^***^ |
| TAM | CCL2 | 0.317 | <0.0001^***^ | 0.243 | <0.0001^***^ |
|  | CD68 | 0.318 | <0.0001^***^ | 0.259 | <0.0001^***^ |
|  | IL10 | 0.330 | <0.0001^***^ | 0.258 | <0.0001^***^ |
| M1 Macrophage | NOS2 | 0.208 | <0.0001^***^ | 0.177 | <0.0001^***^ |
|  | IRF5 | 0.223 | <0.0001^***^ | 0.158 | 0.000^*^ |
|  | PTGS2 | 0.170 | 0.000^**^ | 0.178 | <0.0001^***^ |
| M2 Macrophage | CD163 | 0.409 | <0.0001^***^ | 0.356 | <0.0001^***^ |
|  | VSIG4 | 0.327 | <0.0001^***^ | 0.276 | <0.0001^***^ |
|  | MS4A4A | 0.395 | <0.0001^***^ | 0.341 | <0.0001^***^ |
| Neutrophils | CEACAM8 | 0.378 | <0.0001^***^ | 0.387 | <0.0001^***^ |
|  | CCR7 | 0.404 | <0.0001^***^ | 0.332 | <0.0001^***^ |
|  | ITGAM | 0.463 | <0.0001^***^ | 0.409 | <0.0001^***^ |
| Natural killer cell | KIR2DL1 | 0.004 | 0.991 | -0.042 | 0.356 |
|  | KIR2DL3 | 0.026 | 0.558 | -0.030 | 0.502 |
|  | KIR2DL4 | -0.152 | 0.001^**^ | -0.232 | <0.0001^***^ |
|  | KIR2DS4 | 0.038 | 0.389 | -0.022 | 0.628 |
|  | KIR3DL1 | 0.042 | 0.342 | -0.014 | 0.763 |
|  | KIR3DL2 | 0.047 | 0.290 | -0.030 | 0.504 |
|  | KIR3DL3 | -0.096 | 0.029^*^ | -0.135 | 0.003^*^ |
|  | XCL1 | -0.069 | 0.120 | -0.114 | 0.011 |
|  | CD7 | 0.034 | 0.448 | -0.081 | 0.074 |
| Dendritic cell | HLA-DPA1 | 0.490 | <0.0001^***^ | 0.441 | <0.0001^***^ |
|  | HLA-DPB1 | 0.493 | <0.0001^***^ | 0.442 | <0.0001^***^ |
|  | HLA-DQB1 | 0.363 | <0.0001^***^ | 0.302 | <0.0001^***^ |
|  | HLA-DRA | 0.447 | <0.0001^***^ | 0.392 | <0.0001^***^ |
|  | CD1C | 0.539 | <0.0001^***^ | 0.501 | <0.0001^***^ |
|  | NRP1 | 0.400 | <0.0001^***^ | 0.379 | <0.0001^***^ |
| Th1 | STAT1 | 0.132 | 0.003^**^ | 0.054 | 0.235 |
|  | STAT4 | 0.363 | <0.0001^***^ | 0.285 | <0.0001^***^ |
|  | TBX21 | 0.248 | <0.0001^***^ | 0.159 | 0.000^*^ |
|  | TNF | 0.231 | <0.0001^***^ | 0.140 | 0.002^*^ |
|  | IFNG | -0.031 | 0.480 | -0.129 | 0.004^*^ |
| Th2 | GATA3 | 0.311 | <0.0001^***^ | 0.222 | <0.0001^***^ |
|  | STAT5A | 0.487 | <0.0001^***^ | 0.428 | <0.0001^***^ |
|  | STAT6 | 0.297 | <0.0001^***^ | 0.330 | <0.0001^***^ |
|  | IL13 | 0.126 | 0.004^*^ | 0.073 | 0.102 |
| Th9 | TGFBR2 | 0.698 | <0.0001^***^ | 0.688 | <0.0001^***^ |
|  | IRF4 | 0.326 | <0.0001^***^ | 0.251 | <0.0001^***^ |
|  | PU.1 | 0.382 | <0.0001^***^ | 0.311 | <0.0001^***^ |
| Th17 | STAT3 | 0.354 | <0.0001^***^ | 0.367 | <0.0001^***^ |
|  | IL17A | -0.008 | 0.843433 | -0.060 | 0.181 |
| Tfh | BCL6 | 0.287 | <0.0001^***^ | 0.286 | <0.0001^***^ |
|  | IL21 | 0.074 | 0.095 | 0.021 | 0.637 |
| Th22 | CCR10 | 0.041 | 0.352 | 0.043 | 0.340 |
|  | AHR | 0.463 | <0.0001^***^ | 0.448 | <0.0001^***^ |
| Treg | FOXP3 | 0.308 | <0.0001^***^ | 0.220 | <0.0001^***^ |
|  | CCR8 | 0.402 | <0.0001^***^ | 0.337 | <0.0001^***^ |
|  | TGFB1 | 0.542 | <0.0001^***^ | 0.494 | <0.0001^***^ |
|  | STAT5B | 0.455 | <0.0001^***^ | 0.448 | <0.0001^***^ |
| T cell exhaustion | PDCD1 | 0.142 | 0.001^**^ | 0.027 | 0.544 |
|  | HAVCR2 | 0.354 | <0.0001^***^ | 0.277 | <0.0001^***^ |
|  | CTLA4 | 0.236 | <0.0001^***^ | 0.126 | 0.005^*^ |
|  | ITGAX | 0.346 | <0.0001^***^ | 0.265 | <0.0001^***^ |
|  | LAG3 | 0.049 | 0.270 | -0.056 | 0.215 |
|  | GZMB | -0.082 | 0.064 | -0.205 | <0.0001^***^ |

Note: LUAD, lung adenocarcinoma; ^*^ p<0.05; ^**^p<0.01; ^***^<0.001.

**Table S3. Correlation analysis between FAT4 mRNA expression and methylation sites in TCGA**

| **Methylation sites** | **LUAD** | |
| --- | --- | --- |
|  | Cor | P |
| cg25879360 | 0.3265 | <0.0001^***^ |
| cg26389756 | 0.3617 | <0.0001^***^ |
| cg05118638 | -0.0432 | 0.3582 |
| cg10731073 | -0.0655 | 0.1635 |
| cg03404279 | -0.0262 | 0.5784 |
| cg03527919 | -0.0762 | 0.1049 |
| cg04459504 | -0.0715 | 0.1285 |
| cg08644023 | -0.0717 | 0.1272 |
| cg12828819 | -0.0037 | 0.9376 |
| cg04023369 | 0.0099 | 0.0349^*^ |
| cg18202623 | 0.1089 | 0.0203^*^ |
| cg04373334 | -0.0118 | 0.8029 |
| cg08575049 | 0.0167 | 0.7234 |
| cg17265829 | -0.0413 | 0.3804 |
| cg22911422 | -0.0743 | 0.1139 |
| cg23901852 | -0.0624 | 0.1843 |

Note: LUAD, lung adenocarcinoma; ^*^ *P*<0.05; ^**^ *P* <0.01; ^***^ *P* <0.001.
